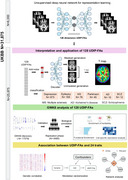# Unveiling the genetic architecture of white matter tracks through unsupervised deep representation learning of fractional anisotropy images

**DOI:** 10.1002/alz70856_101952

**Published:** 2025-12-25

**Authors:** Xingzhong Zhao, Wei He, Ziqian Xie, Myriam Fornage, Degui Zhi

**Affiliations:** ^1^ The University of Texas Health Science Center at Houston, Houston, TX, USA; ^2^ The Brown Foundation Institute of Molecular Medicine, McGovern Medical School, The University of Texas Health Science Center at Houston, Houston, TX, USA

## Abstract

**Background:**

Fractional anisotropy (FA) is a widely used MRI biomarker for assessing the microstructural integrity of white matter (WM) in diffusion MRI studies. It is crucial for understanding neurodevelopment, brain aging, and pathologies like Alzheimer's Disease. Most studies have utilized white‐matter tract atlases (i.e., ICBM‐DTI‐8) to investigate the genetic architecture of FA, but atlas‐based approach has biases by extraction variability and ignores complex interactions between WM tract, limiting their robustness and applicability.

**Method:**

We trained an unsupervised deep neural network using FA images (precomputed from diffusion tensor imaging data) from 6,000 UK Biobank participants (UKBB) to derive a 128‐dimensional representation, named the Unsupervised Deep Learning‐Derived Imaging Phenotypes of FA (UDIP‐FAs). We adopted perturbation‐based Decoder Interpretation and brain disorders classification tasks to evaluate the interpretation and application. Genome‐wide association study (GWAS) was performed on the UDIP‐FAs using 25,875 participants from UKBB. Further analyses involved validation, functional annotation, gene mapping, and exploring genetic associations and causal effects associated with 24 traits.

**Result:**

We discovered that the UDIP‐FAs can be used to broadly classify six distinct brain disorders (AUC: 0.64±0.08). UDIP‐FAs exhibited significantly estimated SNP heritability (*P* < 2.20e‐16, Mann–Whitney U test, mean = 50.81%), higher than that of traditional non‐learning defined FA phenotypes. UDIP‐FA GWAS identified 3782 significant SNPs (*P* < 5e‐8) with 36 lead SNPs, mapped to 156 genes, dubbed UDIP‐FA related genes (UFAGs). These loci showed significant heritability enrichment in oligodendrocyte precursor cells (OPCs, False Discovery Rate (FDR) < 2.88e‐4, Wald Test). Additionally, UFAGs were significantly enriched in previously reported WM‐related gene sets (Bonferroni corrected *P* < 1.12e‐10, Hypergeometric Test), and exhibited significant expression enrichment in glial cells, particularly in OPCs and oligodendrocytes (*P <* 0.03, Wald Test). Notably, we found two UFAGs, ZIC1 and ZIC4, as transcription factors regulated some AD risk genes in brain regulatory network. Moreover, UDIP‐FAs showed significant genetic correlations with intelligence (FDR < 0.03, Wald Test).

**Conclusion:**

Our proposed UDIP‐FAs provide a more unbiased and heritable description of WM, and help unveiling the genetic structure of WM, offering a potentially effective approach to exploring biological mechanism linking WM and brain disorders.